# Bromide supplementation exacerbated the renal dysfunction, injury and fibrosis in a mouse model of Alport syndrome

**DOI:** 10.1371/journal.pone.0183959

**Published:** 2017-09-05

**Authors:** Tsubasa Yokota, Kohei Omachi, Mary Ann Suico, Haruka Kojima, Misato Kamura, Keisuke Teramoto, Shota Kaseda, Jun Kuwazuru, Tsuyoshi Shuto, Hirofumi Kai

**Affiliations:** 1 Department of Molecular Medicine, Graduate School of Pharmaceutical Sciences, Kumamoto University, 5–1 Oe-honmachi, Chuo-ku, Kumamoto City, Kumamoto, Japan; 2 Program for Leading Graduate School “HIGO (Health Life science: Interdisciplinary and Glocal Oriented) Program”, Kumamoto University, 5–1 Oe-honmachi, Chuo-ku, Kumamoto City, Kumamoto, Japan; Temple University, UNITED STATES

## Abstract

A seminal study recently demonstrated that bromide (Br^-^) has a critical function in the assembly of type IV collagen in basement membrane (BM), and suggested that Br^-^ supplementation has therapeutic potential for BM diseases. Because salts of bromide (KBr and NaBr) have been used as antiepileptic drugs for several decades, repositioning of Br^-^ for BM diseases is probable. However, the effects of Br^-^ on glomerular basement membrane (GBM) disease such as Alport syndrome (AS) and its impact on the kidney are still unknown. In this study, we administered daily for 16 weeks 75 mg/kg or 250 mg/kg (within clinical dosage) NaBr or NaCl (control) via drinking water to 6-week-old AS mice (mouse model of X-linked AS). Treatment with 75 mg/kg NaBr had no effect on AS progression. Surprisingly, compared with 250 mg/kg NaCl, 250 mg/kg NaBr exacerbated the progressive proteinuria and increased the serum creatinine and blood urea nitrogen in AS mice. Histological analysis revealed that glomerular injury, renal inflammation and fibrosis were exacerbated in mice treated with 250 mg/kg NaBr compared with NaCl. The expressions of renal injury markers (*Lcn2*, *Lysozyme*), matrix metalloproteinase (*Mmp-12*), pro-inflammatory cytokines (*Il-6*, *Il-8*, *Tnf-α*, *Il-1β*) and pro-fibrotic genes (*Tgf-β*, *Col1a1*, *α-Sma*) were also exacerbated by 250 mg/kg NaBr treatment. Notably, the exacerbating effects of Br^-^ were not observed in wild-type mice. These findings suggest that Br^-^ supplementation needs to be carefully evaluated for real positive health benefits and for the absence of adverse side effects especially in GBM diseases such as AS.

## Introduction

Twenty-seven trace elements had been previously considered essential for the maintenance of the human body [1]. Recently, bromide (Br^-^) was proposed as the 28^th^ essential trace element, and was demonstrated to be critical in the assembly of type IV collagen scaffolds of the basement membrane (BM) [2]. Specifically, Br^-^ is a necessary co-factor for the formation of sulfilimine crosslinks of collagen IV scaffold, which is important for basement membrane and tissue integrity. Sulfilimine crosslink formation, which structurally reinforces the collagen IV scaffold, is catalyzed by peroxidasin, a heme peroxidase [3, 4]. Interestingly, Br^—^deficient *Drosophila* phenotypically copies the peroxidasin mutant *Drosophila*, indicating that both are necessary for collagen IV scaffold production. Br^-^ deficiency in the diet of *Drosophila* led to gross disruption of the BM assembly and tissue development, whereas Br^-^ supplementation resulted in the recovery of the disrupted BM [2]. This study clearly suggested that Br^-^ is essential in the peroxidasin-mediated assembly of collagen IV scaffold, and that Br^-^ supplementation could be a viable adjunctive therapeutic approach to ameliorate BM disorders.

Alport syndrome (AS) is a hereditary, progressive kidney disease caused by GBM disorder [5, 6]. In AS, mutation in the collagen type IV alpha (COL4A) genes *Col4A3*, *A4*, *or A5* eventually leads to GBM degeneration and consequent proteinuria, inflammation and fibrosis, which are phenotypes that are similar to other chronic kidney diseases (CKD) [7]. The absence of one of these type IV collagen genes due to mutation precludes the formation of α3/α4/α5 (Ⅳ) protomers in the GBM that normally replace the α1/α1/α2 (Ⅳ) network during human kidney development. The failure to form α3/α4/α5(Ⅳ) network results in the maintenance of α1/α1/α2 (Ⅳ) in the adult GBM. However α1/α1/α2 (Ⅳ) network is more susceptible to endoproteolysis compared with the more stable and stronger a3/a4/a5(IV) network [8]. The degeneration of α1/α1/α2 (Ⅳ) protomer causes GBM dysfunction and progressive renal failure. Strengthening the α1/α1/α2 (Ⅳ) protomer is one possible approach to retard AS progression [9].

Bromide, in the form of potassium or sodium bromide and triple bromide elixir (sodium, potassium and ammonium salts of bromide), was the first antiepileptic drug used, and is still being used especially in refractory epilepsy cases in children [10]. While effects of Br^-^ in the brain and other organs have been well investigated, its impact on the kidney has not been studied. Here, we investigated the effect of Br^-^ supplementation in X-linked AS mouse model (*G5X*) starting at the early stage, that is, before the onset of proteinuria. Specifically, we evaluated the effect of Br^-^ on the progressive pathological phenotypes in AS mice such as proteinuria, inflammation and fibrosis. Treatment with 75 mg/kg NaBr (administered via drinking water) did not ameliorate the renal dysfunction in AS mice. Surprisingly, 250 mg/kg NaBr exacerbated the renal dysfunction in AS mice. No adverse effects on renal function were observed in wild-type (WT) mice. This study revealed that although Br^-^ is an essential trace element for the formation of BM network, Br^-^ supplementation needs careful evaluation for application to GBM disorders such as AS.

## Materials and methods

### Animals and in vivo treatment

X-linked Alport syndrome mouse model (Col4a5tm1Yseg G5X mutant) was described previously [11]. Mice were obtained from the Jackson laboratory (Bar Harbor, Maine). We used age-matched WT C57BL/6 mice (Charles River Laboratories, Inc.) as control to compare with AS mice. Six-week-old WT and AS male mice were given NaCl- or NaBr-supplemented drinking water (NaCl or NaBr lower dose: 75 mg/kg/day; higher dose 250 mg/kg/day). High-grade NaCl and NaBr were purchased from Nacalai Tesque (Kyoto, Japan). The chow fed to the animals was Br^-^ free. NaCl- or NaBr-treated AS mice were sacrificed at 22 weeks old, and kidney tissues and sera were collected. NaCl- or NaBr-treated WT mice were sacrificed at 32 weeks old to assess the long-term effect of Br^-^. All animal experiments were approved by the Animal Care and Use committee of Kumamoto University (#A28-059).

### Proteinuria score

Urine collection was performed using metabolic cage (AS ONE Corporation) for 24 hr once every 4 weeks. Urinary protein and creatinine were measured by Bradford method (Bio-Rad Laboratories) and Jaffe’s method (Wako Pure Chemical Industories), respectively. Urinary protein concentration was normalized with urinary creatinine concentration, and presented as proteinuria score.

### Serum creatinine and blood urea nitrogen

Mouse blood was collected from abdominal aorta. Fresh blood samples were centrifuged at 800 g, 4 ^o^C, 15 min, and blood plasma was isolated. Blood urea nitrogen (BUN) of plasma was measured using Fuji Dri Chem BUN PⅢ (Fujifilm, Japan). Serum creatinine was measured by Jaffe’s method (Wako Pure Chemical Industries, Japan). The evaluation of samples was carried out according to the manufacturer’s recommended protocol.

### Evaluation of glomerular injury score, inflammatory cell infiltration and tubulointerstitial fibrosis score

For renal histology, mouse kidneys were fixed in 10% formalin and embedded in paraffin for Periodic Acid-Schiff (PAS), H&E staining and Masson-Trichrome (MT) staining. Tissue blocks were sliced into 4-μm thickness. Glomerular injury score, inflammatory cell infiltration, and tubulointerstitial fibrosis score were assessed as described previously [12]. Briefly, for assessment of glomerular injury, renal sections were stained with PAS. To evaluate the glomerular injury score, more than 50 PAS-stained random glomeruli per mouse (n = 4 mice) were examined, and scored from 0 to 4 (0, no lesion; 1, expansion of mesangial area; 2, expansion of Bowman’s epithelial cells and adhesion of glomeruli and Bowman’s capsule; 3, sclerotic area in 50% - 75% of glomerulus; 4, sclerotic area in 75% - 100% of glomerulus). Double blind scoring was performed and values were computed and presented in a graph as percentage. For tubulointerstitial fibrosis score, MT-stained kidneys were evaluated using Bio-Revo imaging and analysis software (Keyence, Japan). MT-stained area vs unstained area was calculated and presented as % fibrotic region.

### Immunohistochemistry

For immunohistochemistry, kidney tissues were immersed consecutively in 10% formalin and 75% ethanol, and embedded in paraffin. De-paraffinization step was done with xylene and ethanol. Antigen activation was performed using Dako proteinase K for 15 min or Dako real target retrieval solution (Dako, Japan) at 98°C for 40 min, then samples were reacted with F4/80 (#6640, Abcam) or type Ⅳ collagen (#6586, Abcam) diluted at 1:100. Histofine Simple stain (Nichirei Biosciences Inc., Japan) was used for secondary antibody reactions. After TBS wash, DAB reaction was performed for 1–10 min. Slides were stained with Haematoxylin for 60 sec. F4/80 and type Ⅳ collagen were evaluated using Bio-Revo imaging and analysis software (Keyence, Japan).

### Real-time quantitative RT-PCR analysis (Q-RT-PCR)

Total RNA was isolated with RNAiso reagent (Takara Bio Inc.) following the manufacturer’s instructions. Reverse transcription and PCR amplifications were performed as described previously [13]. The sequences of primers used for Q-RT-PCR are shown in Table 1.

**Table 1:** Primer sequence for real-time quantitative RT-PCR.

Gene	Sense	Antisense
*Lcn2*	5'- GAGAAGGCAGCTTTACGATG -3'	5'-CCTGGAGCTTGGAACAAATG -3'
*Lysozyme*	5'-CCAGTGTCACGAGGCATTCA-3'	5'-TGATAACAGGCTCATCTGTCTCA-3'
*Il-6*	5'-GAGGATACCACTCCCAACAGACC-3'	5'-AAGTGCATCATCGTTGTTCATACA-5'
*Il-8 (KC)*	5'-TGTCAGTGCCTGCAGACCAT-3'	5'-GAGCCTTAGTTTGGACAGGATCTG-3'
*Tnf-α*	5'-CATCTTCTCAAAATTCGAGTGACAA-3'	5'-TGGGAGTAGACAAGGTACAACCC-3'
*Il-1β*	5'-GCTGAAAGCTCTCCACCTCAATG-3'	5'-TGTCGTTGCTTGGTTCT CCTTG-3'
*Mmp12*	5'-CATGAAGCGTGAGGATGTAGAC-3'	5'-TGGGCTAGTGTACCACCTTTG-3'
*Tgf-β*	5'-CACCTGCAAGACCATCGACAT-3'	5'-GAGCCTTAGTTTGGACAGGATCTG-3'
*Col1a1*	5'-CTGGCGGTTCAGGTCCAAT-3'	5'-TTCCAGGCAATCCACGAGC-3'
*α-Sma*	5'-CCCAGACATCAGGGAGTAATGG-3'	5'-TCTATCGGATACTTCAGCGTCA-3'
*Bmp7* *Peroxidasin*	5'-GCGCCTCTGTTCTTGCTGCG-3' 5'-GACAGGCAAGCATTTAAGGGA-3'	5'-GGCGCCGGTGGATGAAGCTG-3' 5'-TCCAATCGCAGCCGTTTCAT-3'
*Gapdh*	5'-CCTGGAGAAACCTGCCAAGTATG-3'	5'-GGTCCTCAGTGTAGCCCAAGATG-3'

### Western blotting analysis

Isolated whole kidneys were lysed in radioimmunoprecipitation (RIPA) buffer and subjected to Western blot analysis. Immunoblots were probed with type IV collagen (#6586; Abcam) and phosphorylated Smad1/5/8 (#95115; Cell Signaling Technology) antibodies followed by their respective HRP-conjugated secondary antibodies. γ-Tubulin (sc-7396; Santa Cruz Biotechnologies, Inc.) was used as loading control. SuperSignal WestPico chemiluminescence substrate (Thermo Scientific Inc.) was used for visualizing the blots.

### Measurement of Br^-^ concentration in kidney tissues

Measurement of Br^-^ concentration in the kidney tissues was performed by Shimadzu Techno-Research, Inc. Briefly, 0.1 gram of kidney tissue was collected from 3 to 5 mice in the same group. Kidney tissues were homogenized in ultrapure water (0.2 mL). Mixed solution (ultrapure water and ethanol, 1:1) was added, and sample weight was measured. Samples were burnt at inlet 900°C / outlet 1000°C. Br^-^ was trapped from kidney sample as alkaline solution (1 mol/L NaOH 2 mL, H_2_O_2_ 100 μl and hydrazine monohydrate 50 μl (total 500ml)), and Br^-^ was measured by ICP-MS. ICP-MS measuring conditions are shown in Table 2.

**Table 2:** ICP-MS measuring conditions for Br^-^.

Instrument	ICP-MS, 7700x (Agilent Technologies, Inc)
RF power	1550 W
Plasma gas flow	15.0 L/min
Auxiliary gas flow	0.9 L/min
Carrier gas flow	1.0 L/min
Sampling depth	10 mm
Collision / Reaction gas	H_2_ 6mL/min
Integration time	1 sec
Sweep	100 times per integration
Repeat integration	3 times
m/z	79 (Br) / 89 (Y, internal standard)

### Statistical analysis

All data are presented as mean ± S.D. Significance of the difference between groups was assessed using analysis of variance (ANOVA) with Tukey-Kramer test. A *P* value of <0.05 is considered statistically significant.

## Results

### NaBr exacerbates the renal dysfunction in AS mice

**Fig 1:**
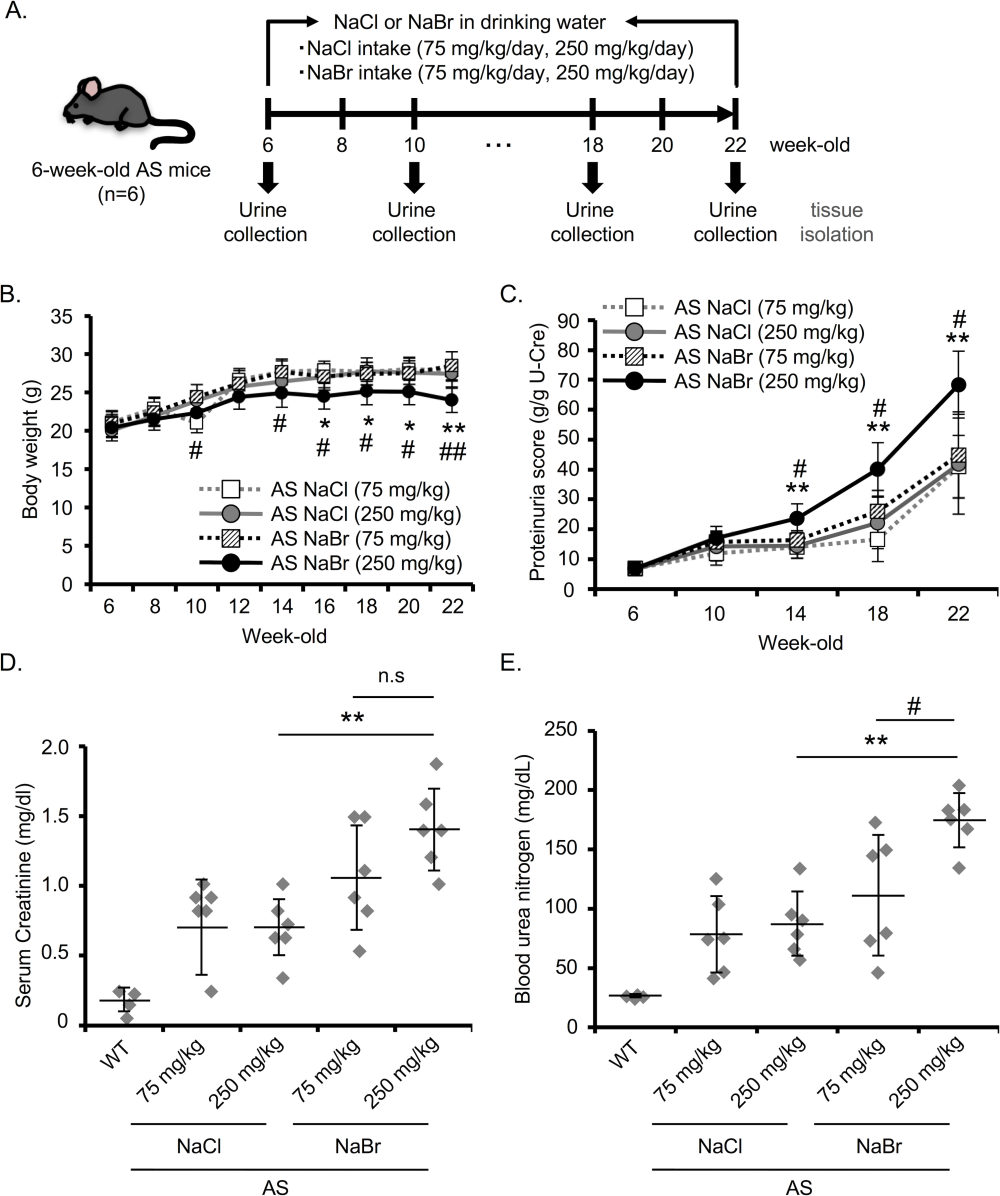
NaBr exacerbates the renal dysfunction in AS mice. (A) Schematic diagram of the experimental design for NaCl or NaBr treatment in AS mice. (B) Body weight was measured every two weeks. (C) Urine samples were collected every four weeks, and urinary protein was measured by Bradford method. Proteinuria score was calculated based on urinary protein and creatinine concentrations in NaCl- or NaBr-treated AS mice (mean±S.D. n = 6). (D) Serum creatinine was measured by Jaffe’s method in WT, NaCl- or NaBr-treated AS mice at 22 weeks. (E) BUN score of each group in 22-week-old mice was measured. Bars indicate the mean ± S.D. *P<0.05; **P<0.01 versus NaCl (250 mg/kg)-treated AS; #P<0.05; ##P<0.01 versus NaBr (75 mg/kg)-treated AS mice. P values were assessed by Tukey-Kramer test. n.s., not significant.

To investigate the effect of Br^-^ on AS pathology, 6-week-old AS mice were administered with 75 mg/kg/day or 250 mg/kg/day NaCl or NaBr via drinking water (Fig 1A). These doses were chosen based on the clinically used dose of Br^-^ [14, 15]. LD_50_ (median lethal dose) of Br^-^ for mice is 5020 mg/kg body weight [16]. NaCl was used as control for the intake of sodium ion. No significant difference in the urine volume was observed among the groups (S1A Fig). We found that 250 mg/kg NaBr slightly but statistically decreased the body weight of AS mice (Fig 1B) although no statistical difference in food intake was observed among the groups (S1B Fig). 75 and 250 mg/kg NaCl and 75 mg/kg NaBr had no significant differences in proteinuria score. However, 250 mg/kg NaBr exacerbated the progressive proteinuria starting in 14-week-old mice (Fig 1C). 250 mg/kg NaBr also significantly elevated the serum creatinine level compared with high dose NaCl in 22-week-old AS mice (Fig 1D). We next assessed the blood urea nitrogen (BUN) in 22-week-old AS mice. BUN was increased in 250 mg/kg NaBr-treated AS mice compared with NaCl- and 75 mg/kg NaBr-treated AS mice (Fig 1E). These data indicate that high dose NaBr worsens the renal dysfunction in AS.

### NaBr exacerbates the glomerular injury and the expression of renal injury markers in AS mice

**Fig 2:**
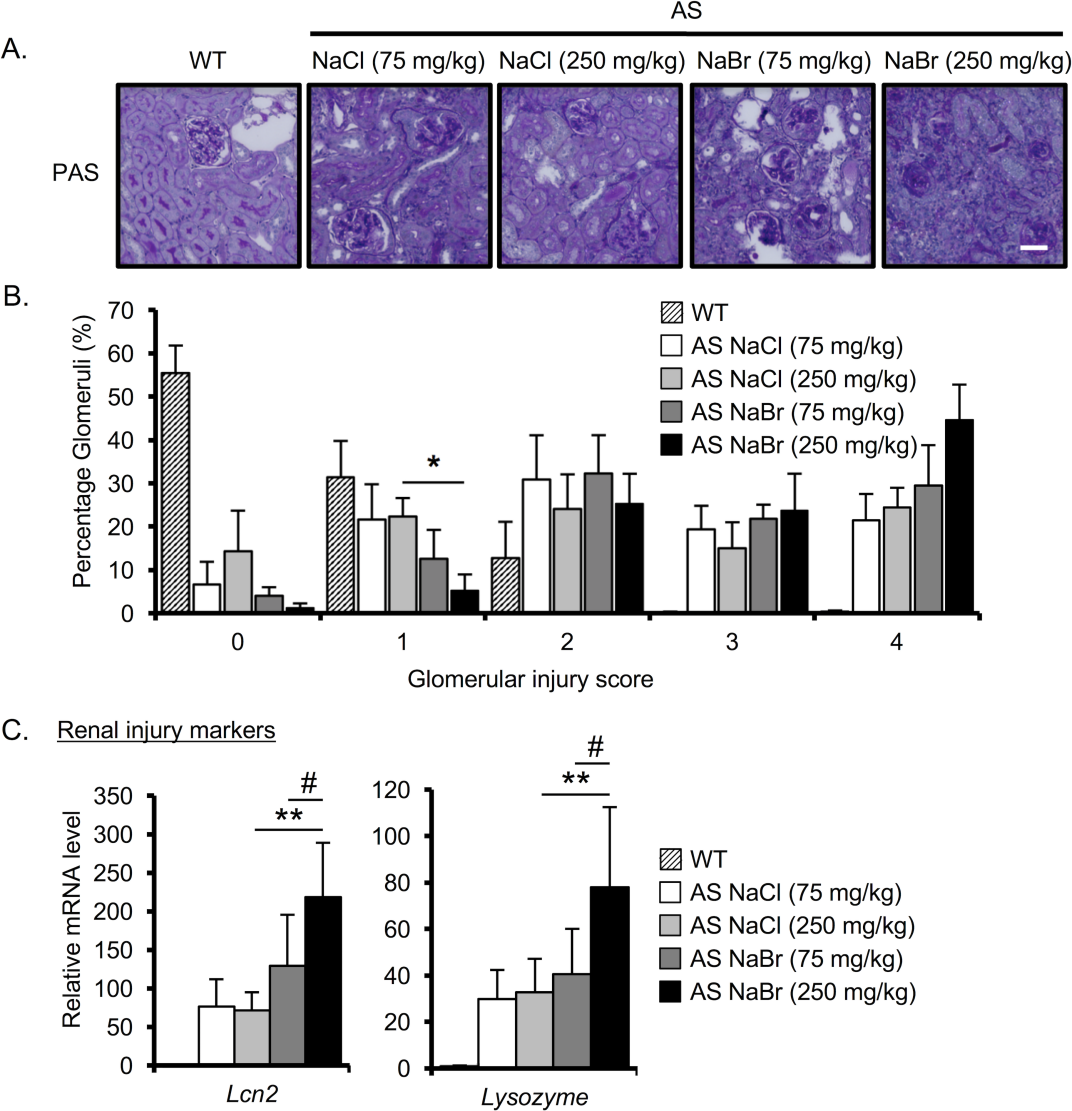
NaBr exacerbates the glomerular injury and increases the expression of renal injury markers in AS mice. (A) Representative images of PAS-stained renal sections from 22-week-old mice AS mice and age-matched WT mice. Scale bar, 50 μm. (B) Glomerular injury score was evaluated based on the PAS-stained sections. (C) Total RNA was isolated from renal tissues of 22-week-old mice. Quantitative RT-PCR was performed to analyze the expression of the indicated renal injury markers such as *Lcn2* and *Lysozyme*. The data were normalized to *Gapdh*. Bars indicate the mean ± S.D. (n = 4–6). *P<0.05; **P<0.01 versus NaCl (250 mg/kg)-treated AS mice; #P<0.05; versus NaBr (75 mg/kg)-treated AS mice. P values were assessed by Tukey-Kramer test.

To clarify the effect of Br^-^ on the histopathology in AS mice, we stained kidney sections with PAS. We assessed the glomerular injury, a characteristic of AS pathology (Fig 2A). In WT mice, more than 50% of the PAS-stained glomeruli had 0 severity score (Fig 2B; see Methods for glomerular injury score). As expected, AS mice at 22 weeks had varying severity of glomerular injury, and only less than 15% of glomeruli were lesion-free. Notably, AS mice treated with 250 mg/kg NaBr had the highest percentage (>40%) of glomeruli that displayed severe glomerular injury (score of 4) with very few lesion-free glomeruli. Although the percentage of glomeruli that displayed a score of 4 in 250 mg/kg NaBr-treated AS mice was not statistically significant compared with 250 mg/kg NaCl-treated AS mice, the percentage of glomeruli that had a severity score of 1 (low severity) was statistically decreased in 250 mg/kg NaBr-treated AS mice compared with 250 mg/kg NaCl-treated AS mice (Fig 2B). We next investigated the effect of Br^-^ on the expression of renal injury markers such as *Lcn2* and *Lysozyme*. Q-RT-PCR analysis revealed that the expression of *Lcn2* and *Lysozyme* was up-regulated in 250 mg/kg NaBr-treated AS mice compared with NaCl-treated mice (Fig 2C) Taken together, Br^-^ exacerbates the glomerular injury and increases the expression of renal injury markers.

### NaBr enhances the renal inflammation in AS mice

**Fig 3:**
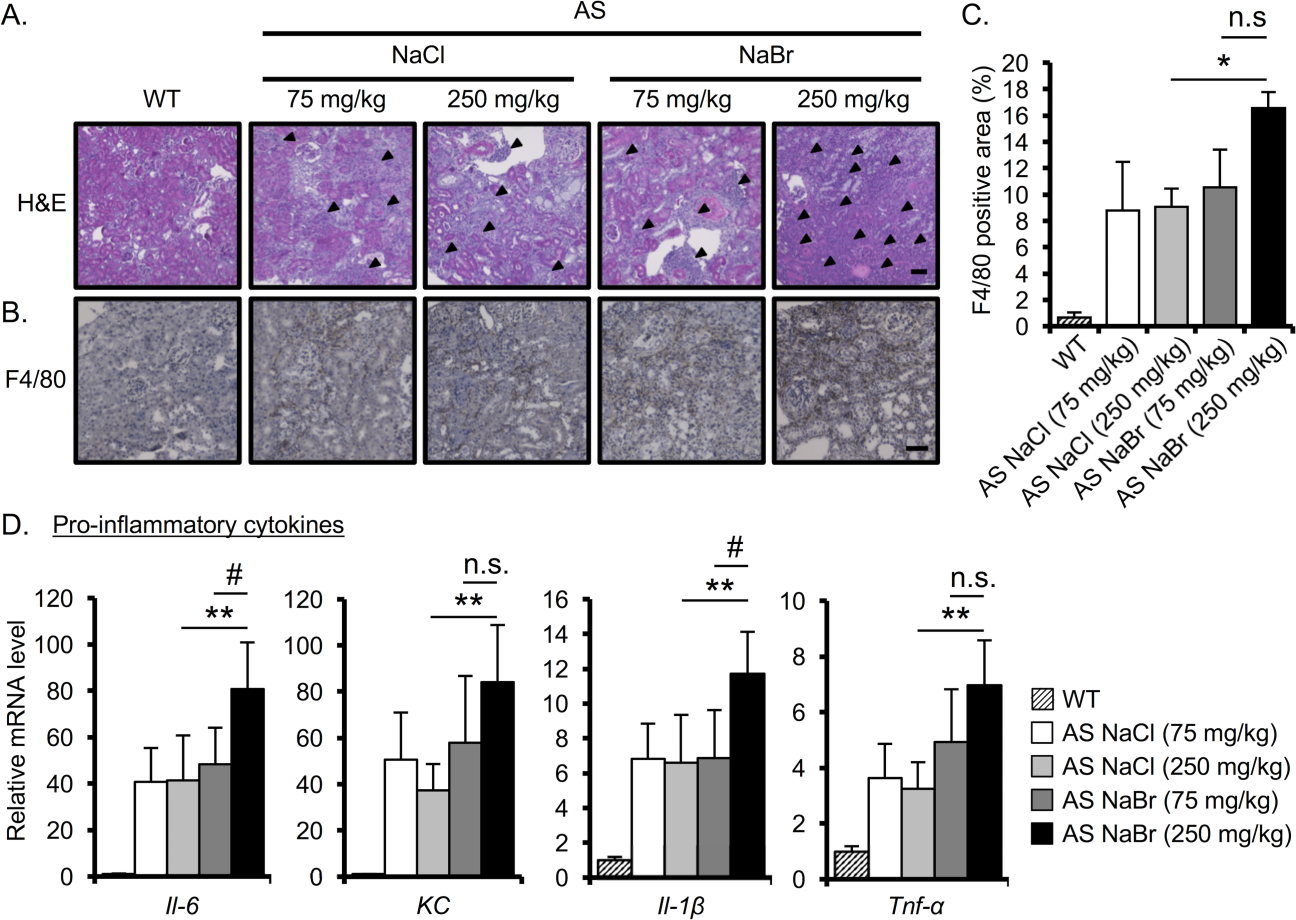
NaBr exacerbates the renal inflammation. (A-B) Kidney sections of 22-week-old mice were stained with H&E (A) or immunostained with F4/80 (B). Scale bar, 50 μm. (C) F4/80-positive area was quantified based on the immunostained images using Bio-Revo imaging and analysis software. (D) Total RNA was isolated from renal tissues of 22-week-old mice. Quantitative RT-PCR was performed to analyze the expression of the indicated pro-inflammatory cytokines. The data were normalized to *Gapdh*. Bars indicate the mean ± S.D. (n = 4–6). *P<0.05; **P<0.01 versus NaCl (250 mg/kg)-treated AS mice; #P<0.05; versus NaBr (75 mg/kg)-treated AS mice. P values were assessed by Tukey-Kramer test. n.s., not significant.

We next assessed the renal inflammation, which is a characteristic pathology of AS. H&E staining showed that inflammatory cell infiltration is increased in 250 mg/kg NaBr-treated AS mice compared with 75 mg/kg and 250 mg/kg NaCl-treated AS mice (Fig 3A). Moreover, to investigate the macrophage infiltration, we stained kidney tissues with macrophage marker (F4/80). We found that 250 mg/kg NaBr treatment enhances F4/80-positive area compared with NaCl treatment (Fig 3B and 3C). These data indicated that 250 mg/kg NaBr induces inflammatory cell invasion. Next, we determined the effect of Br^-^ on the mRNA expression of pro-inflammatory cytokines, which were recently reported to be up-regulated [17, 18]. There was no notable difference in the expression of these genes between 75 mg/kg and 250 mg/kg NaCl as well as with 75 mg/kg NaBr treatment (Fig 3D). Importantly, compared with 75 mg/kg NaBr, 250 mg/kg NaBr significantly increased the mRNA expression level of pro-inflammatory cytokines. Taken together, 250 mg/kg NaBr enhances the renal inflammation in AS mice.

### NaBr exacerbates the renal fibrosis in AS mice

**Fig 4:**
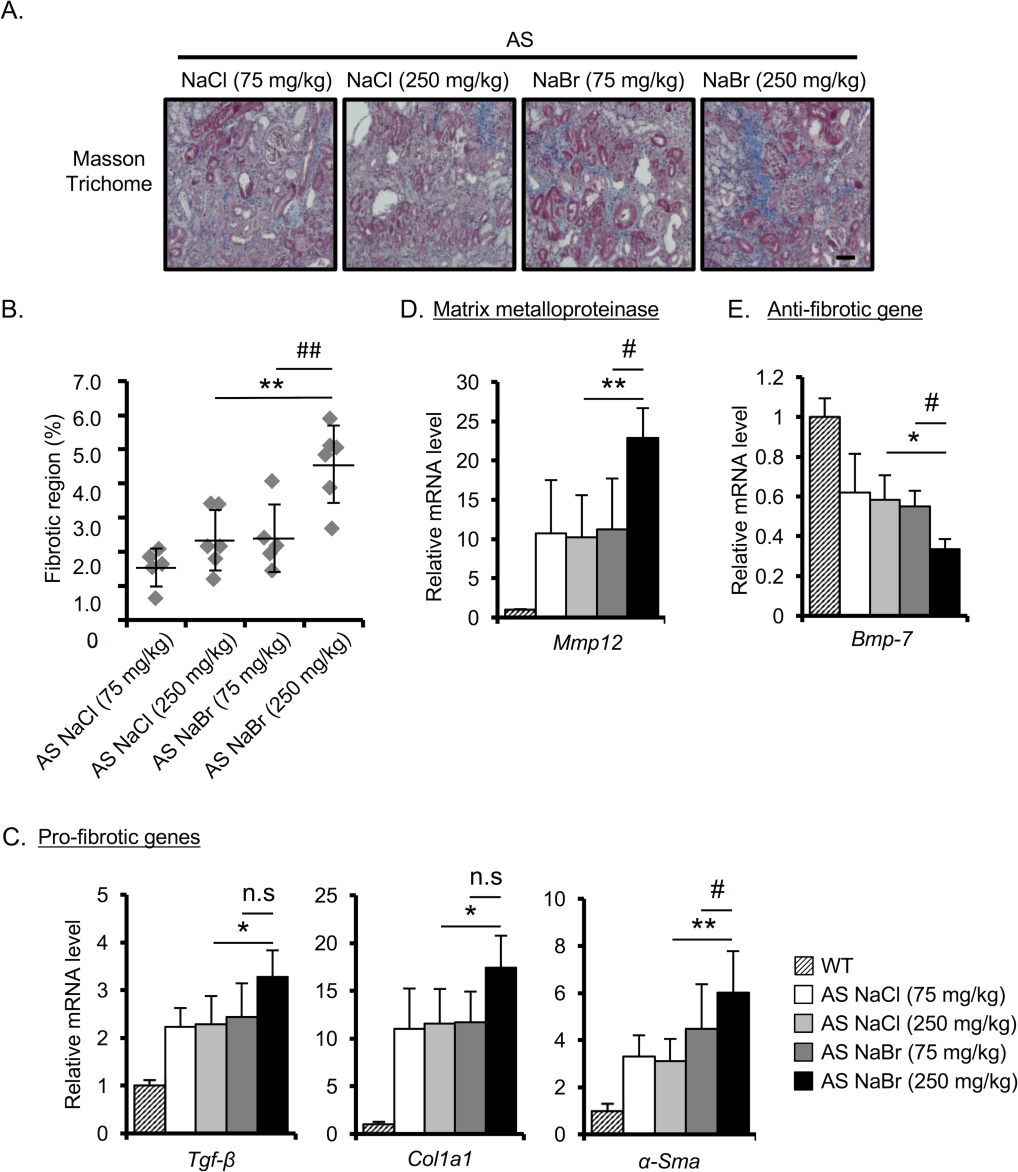
NaBr exacerbates the renal fibrosis in AS mice. (A) Representative images of Masson-Trichrome (MT)-stained renal sections from 22-week-old AS mice are shown. Scale bar, 50 μm. (B) Tubulointerstitial fibrosis score was evaluated by measuring the fibrotic region in MT-stained section. (C-E) Total RNA was isolated from renal tissues of 22-week-old mice. Quantitative RT-PCR was performed to analyze the expression of the indicated pro-fibrotic genes (C), matrix metalloproteinase (D) and anti-fibrotic genes (E). The data were normalized to *Gapdh*. Bar graphs indicate the mean ± S.D. (n = 4–6). *P<0.05; **P<0.01 vs NaCl (250 mg/kg)-treated AS, #P<0.05; ##P<0.01 versus NaBr (75 mg/kg)-treated AS mice. P values were assessed by Tukey-Kramer test. n.s., not significant.

**Fig 5:**
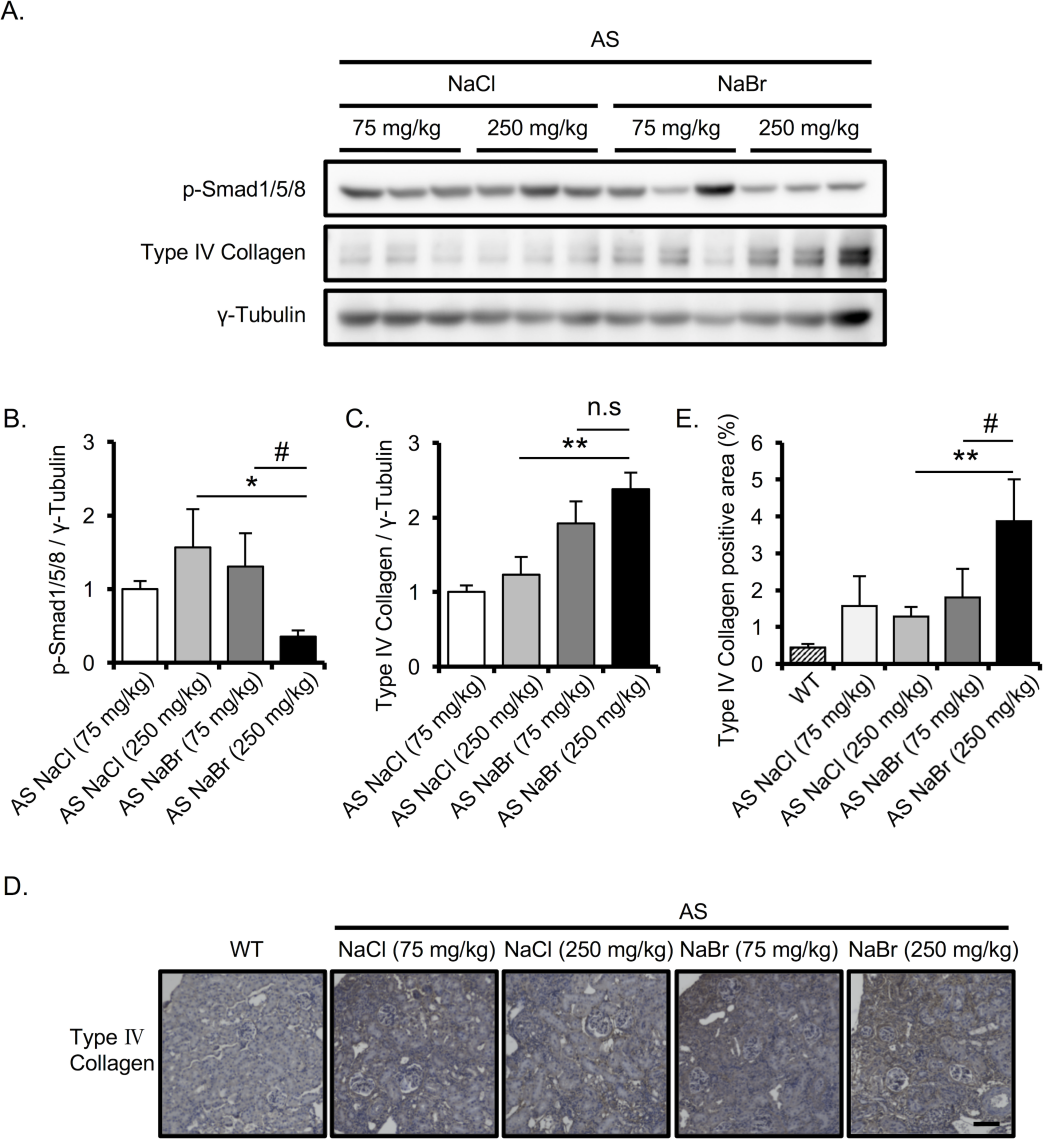
NaBr induces the accumulation of type IV collagen. (A) Lysates were extracted from mouse kidneys of the indicated groups, and subjected to immunoblotting analysis. Protein expressions of type IV collagen, phosphorylated Smad1/5/8 and γ-tubulin were visualized by chemiluminescence. γ-Tubulin was used as loading control. (B-C) Blot intensities were quantified using Image J software (Fujifilm, Japan). Values were normalized to γ-tubulin and presented as relative expression. (D) Kidney sections of 22-week-old mice were immunostained with type Ⅳ collagen. Scale bar, 100 μm. (E) Type Ⅳ collagen-positive area was quantified based on the immunostained images using Bio-Revo imaging and analysis software. Bars indicate the mean ± S.D. (n = 3–4). *P<0.05; **P<0.01 versus NaCl (250 mg/kg)-treated AS, #P<0.05 versus NaBr (75 mg/kg)-treated AS mice. P values were assessed by Tukey-Kramer test. n.s., not significant.

To clarify the effect of Br^-^ on renal fibrosis, which is one of the hallmarks of AS [19], we performed MT staining to measure the fibrotic region. MT staining revealed that 250 mg/kg NaBr significantly enhances the fibrotic region compared with 75 mg/kg and 250 mg/kg NaCl and 75 mg/kg NaBr (Fig 4A and 4B). We next checked the expression of pro-fibrotic markers in AS mice kidney using Q-RT-PCR analysis. Consistent with previous findings, the pro-fibrotic genes and *Mmp-12* were increased in AS mice compared with WT mice (Fig 4C and 4D) [12, 18]. Interestingly, we found that 250 mg/kg NaBr treatment exacerbates the expression of pro-fibrotic genes (*Tgf-β*, *Col1a1*, *α-Sma*) and *Mmp12* compared with NaCl treatment in AS mice (Fig 4C). Moreover, we observed that bone morphogenic protein (*Bmp*)*-7*, an anti-fibrotic protein [20], was down regulated in AS mice, and the *Bmp-7* suppression was exacerbated in 250 mg/kg NaBr-treated AS mice compared with that in 250 mg/kg NaCl-treated AS mice (Fig 4E). Consistently, the protein expression level of phosphorylated Smad1/5/8 (p-Smad), which are the signaling molecules downstream of *Bmp7* [21], was also clearly decreased in the kidney of AS mice treated with 250 mg/kg NaBr (Fig 5A and 5B). Moreover, the expression of type IV collagen, which is one of the markers of fibrosis [22, 23], was increased in renal tissues of AS mice treated with 250 mg/kg NaBr as assessed by western blotting and immunohistochemistry (Fig 5A and 5C–5E). The suppression of *Bmp-7* and p-Smad1/5/8 molecules, which negatively regulate TGF-β signaling [21, 24–26], and the increase of type IV collagen expression in 250 mg/kg NaBr-treated AS mice are consistent with the enhancement of pro-fibrotic factor *Tgf-β* expression (Fig 4C) and the induction of kidney fibrosis (Fig 4A and 4B). We also confirmed that the p-Smad1/5/8 expression was decreased in AS mice kidney compared with that in WT mice, and that the protein expression level of type IV collagen was higher in AS mice than in WT mice (S2A–S2C Fig).

### Long-term NaBr treatment does not affect the renal function in WT mice

**Fig 6:**
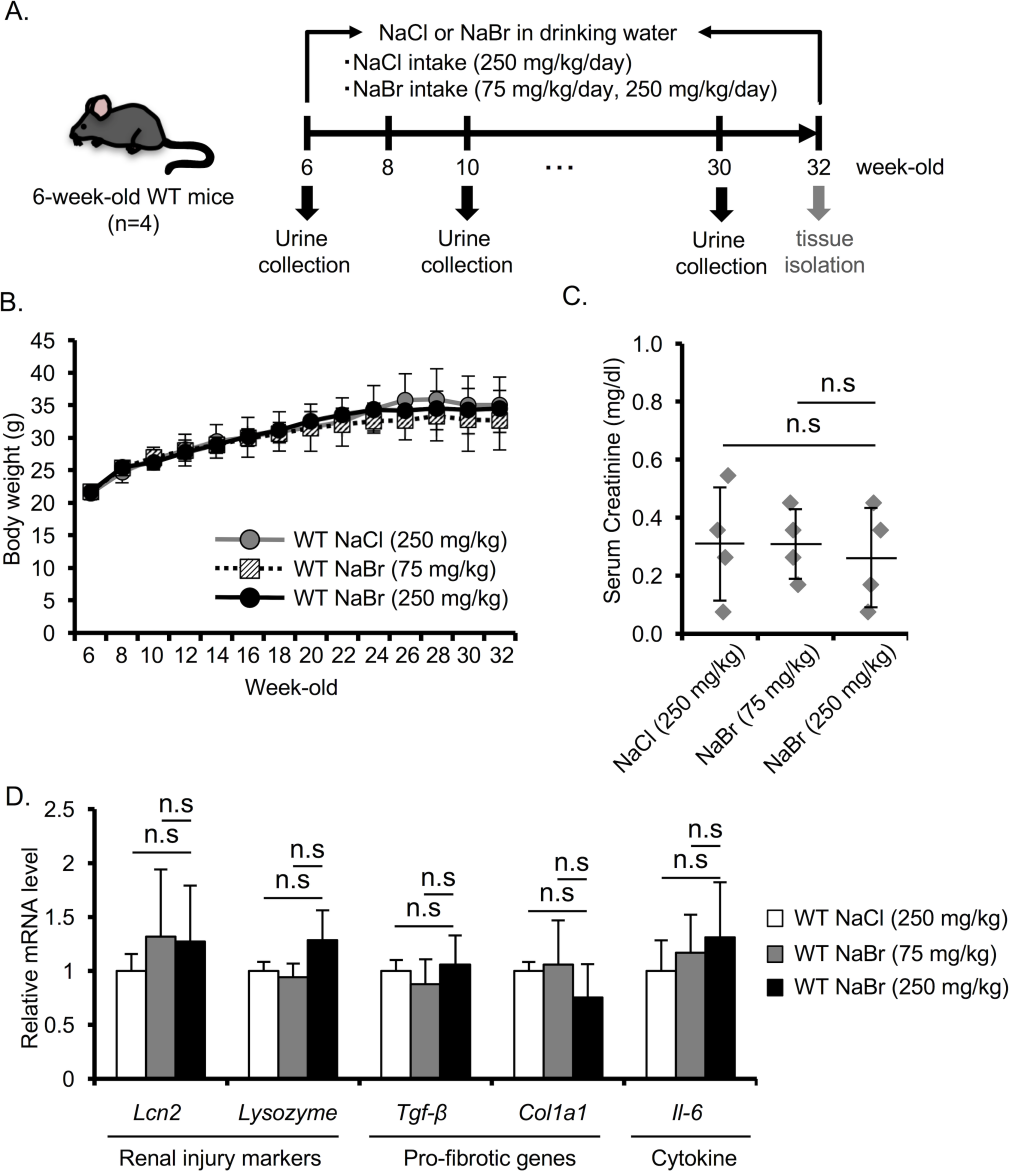
Long-term NaBr treatment does not affect the renal function in WT mice. (A) Schematic diagram of the experimental design for NaCl (250 mg/kg) or NaBr (75 mg/kg, 250 mg/kg) treatment in WT mice. (B) Body weight was measured every two weeks until mice were 32 weeks old. (C) Serum creatinine in 32-week-old mice was measured. (D) Total RNA was isolated from renal tissues of 32-week-old mice. Quantitative RT-PCR was performed to evaluate the expression of the indicated genes. Bars indicate the mean ± S.D. (n = 4). P values were assessed by Tukey-Kramer test. n.s., not significant.

To determine the effect of long-term treatment of Br^-^ on healthy mice, we treated 6-week-old C57BL/6 WT mice with 75 mg/kg and 250 mg/kg NaBr for 26 weeks (Fig 6A). Body weight was measured every 2 weeks until the end of the treatment. 250 mg/kg NaBr administration did not affect the body weight in WT mice (Fig 6B). 250 mg/kg NaBr treatment also did not affect the urine volume and food intake (S3A and S3B Fig). The serum creatinine was not increased by 250 mg/kg NaBr treatment in WT mice (Fig 6C). To determine whether 250 mg/kg NaBr impacts on the expression of renal injury markers, pro-fibrotic genes and pro-inflammatory cytokines in WT mice, we checked the mRNA level of these genes. The expression levels of renal injury markers, pro-fibrotic genes and pro-inflammatory cytokines did not statistically differ in 75 mg/kg and 250 mg/kg treated-NaBr from that in NaCl-treated mice (Fig 6D). Taken together, these results suggest that Br^-^ exacerbates the renal dysfunction in AS but has no adverse effects on the renal functions in WT mice.

## Discussion

Many essential trace elements have significant impact on human health. Too much or too little may lead to the onset of diseases. A highly significant study by McCall *et al*. showed that Br^-^ has a critical function in the assembly of type IV collagen scaffolds, and a deficiency of Br^-^ in the diet led to the dysfunction of the BM network in *Drosophila* [2]. The authors proposed that Br^-^ could be considered as an essential trace element, and their findings suggested that Br^-^ supplementation has potential therapeutic application. Adding value to this proposal is that clinically, end-stage renal disease (ESRD) patients have low plasma Br^-^ due to Br^-^ loss during dialysis [27, 28], and this could negatively affect the amelioration of renal dysfunction. Because Br^-^ is already clinically used as an adjunctive antiepileptic drug, its repositioning for BM diseases is possible.

In this study, we evaluated the effect of Br^-^ supplementation on AS. We reasoned that before the onset of the disease, supplementation with Br^-^ could improve or strengthen the collagen type IV protomer network in the GBM and retard the progression of AS disease. Contrarily, 250 mg/kg NaBr exacerbated the pathological phenotypes in AS mice while 75 mg/kg NaBr neither improved nor worsened AS pathologies. Interestingly, these results were not observed in WT mice. Br^-^ is normally excreted from the kidneys [29]. However, in CKD patients, the estimated glomerular filtration rate (eGFR) decreases as the disease progresses, and urinary Cl^-^ and K^+^ excretion is decreased in CKD patients [30]. The excretion of Br^-^ in CKD patients has not been studied, but because Cl^-^ and Br^-^ are metabolized in the body and excreted similarly in normal kidneys [29], it is likely that Br^-^ excretion is decreased in diseased kidneys analogously to Cl^-^. In this study, we firstly showed that Br- excretion is decreased in AS mice compared with WT mice (Table 3). The accumulation of Br^-^ in the kidneys, made more abundant due to supplementation, could have caused the faster decline of kidney function. The mechanism of how Br^-^ exacerbated the AS pathology is still unclear. McCall et al demonstrated that Br^-^ is used to catalyze the peroxidasin-mediated sulfilimine crosslink formation in collagen IV [2]. While peroxidasin is indispensable for the synthesis of basement membranes [31], it has also been implicated in the formation of fibrotic kidneys [32]. In AS patients, the failure to form a3/a4/a5(IV) network results in the maintenance of a1/a1/a2(IV) in the adult GBM. COL4A1 or COL4A2 acts as component of GBM in glomeruli, which protects from proteinuria leakage, but as a fibrotic factor in tubulointerstitium. We showed that type Ⅳ collagen is accumulated in tubulointerstitium in mice treated with 250 mg/kg NaBr (Fig 5D and 5E). Although we did not examine the activity of peroxidasin in this study, we found that the mRNA expression of peroxidasin was slightly increased in 12-week-old AS mice compared with WT mice and significantly elevated at late-stage AS (22 weeks) (S4A and S4B Fig). Importantly, treatment with 250 mg/kg Br^-^ further enhanced the peroxidasin gene expression (S4B Fig). It is possible that Br^-^ mediates the assembly of type IV collagen by regulating the peroxidasin activity, which could exacerbate the AS pathologies including renal fibrosis. The dampening of the *Bmp-7* and Smad1/5/8 signaling molecules in AS mice treated with 250 mg/kg NaBr together with the increase of *Tgf-β* mRNA and type IV collagen protein expression confirmed the dysregulation of the inhibitory pathway for fibrogenesis. These data suggest that 250 mg/kg NaBr also influences the TGF-β-induced fibrosis. How high dose NaBr exerts this effect and where peroxidasin is located in this signaling network remains to be elucidated. Further investigations may clarify the involvement of peroxidasin in Br^—^mediated exacerbation of kidney fibrosis and dysfunction in AS.

**Table 3:** Bromide ion concentration.

Sample name	Br concentration (in kidney tissue)
AS NaCl (75mg/kg/day)	8 μg/g
AS NaCl (250 mg/kg/day)	7.5 μg/g
AS NaBr (75 mg/kg/day)	190 μg/g
AS NaBr (250 mg/kg/day)	560 μg/g
WT NaBr (250 mg/kg/day)	220 μg/g

The dosage of Br^-^ is important for the risk-benefit balance in the treatment of epilepsy, not least because of the adverse effects of excess Br^-^ and its very long half-life, which is estimated to be 8–14 days in adults and 6–8 days in children [10, 33]. In our study, we used two doses that were reported to be clinically used for epilepsy [14, 15]. The lower dose (75 mg/kg) Br^-^ neither ameliorated nor exacerbated the AS pathology indicating that at low dose, Br^-^ could be tolerated even in CKD condition. Higher dose (250 mg/kg) Br^-^ was toxic in AS mice but not in normal mice suggesting that accumulation of Br^-^ can occur in diseased kidney to exacerbate a pre-existing kidney dysfunction. Because Br^-^ levels are decreased in ESRD patients on dialysis [27], the supplementation of Br^-^ was proposed to benefit ESRD patients. However, according to our data, we need to consider whether Br- supplementation is beneficial for ESRD patients.

## Supporting information

S1 Fig Urine volume and food intake in AS mice. (A, B) Urine volume and food intake were measured every four weeks using metabolic cages for 24 hr.
(TIF)

S2 Fig Type IV collagen is increased and p-Smad1/5/8 is decreased in AS kidney. (A) Whole kidney protein lysates were isolated from 16-week-old WT and AS mice. Type IV collagen and phosphorylated Smad1/5/8 expression was analyzed by immunoblotting. (B) Immunoblots were quantified using Image Gauge software (Fujifilm), normalized to γ-tubulin and presented as relative expression. P values were assessed by unpaired *t*-test.
(TIF)

S3 Fig Urine volume and food intake in WT mice. (A, B) Urine samples and food intake were measured every four weeks until mice were 30 weeks old.
(TIF)

S4 Fig The expression of peroxidasin in middle- or late-stage AS mice. (A) Total RNA was isolated from renal tissues of 12-week-old (middle stage) WT and AS mice. Quantitative RT-PCR was performed to analyze the expression of *Peroxidasin*. (B) Total RNA was isolated from renal tissues of the indicated 22-week-old WT or (late stage) AS mice and quantitative RT-PCR analysis was performed. The data were normalized to *Gapdh*. Bars indicate the mean ± S.D. (n = 3–6). *P<0.05; **P<0.01, n.s., not significant.
(TIF)
