# Supplementary figures and images for: Bromide supplementation exacerbated the renal dysfunction, injury and fibrosis in a mouse model of Alport syndrome
Source: PLoS One. 2017 Sep 5;12(9):e0183959. doi: 10.1371/journal.pone.0183959 (PMC5584969; doi:10.1371/journal.pone.0183959)

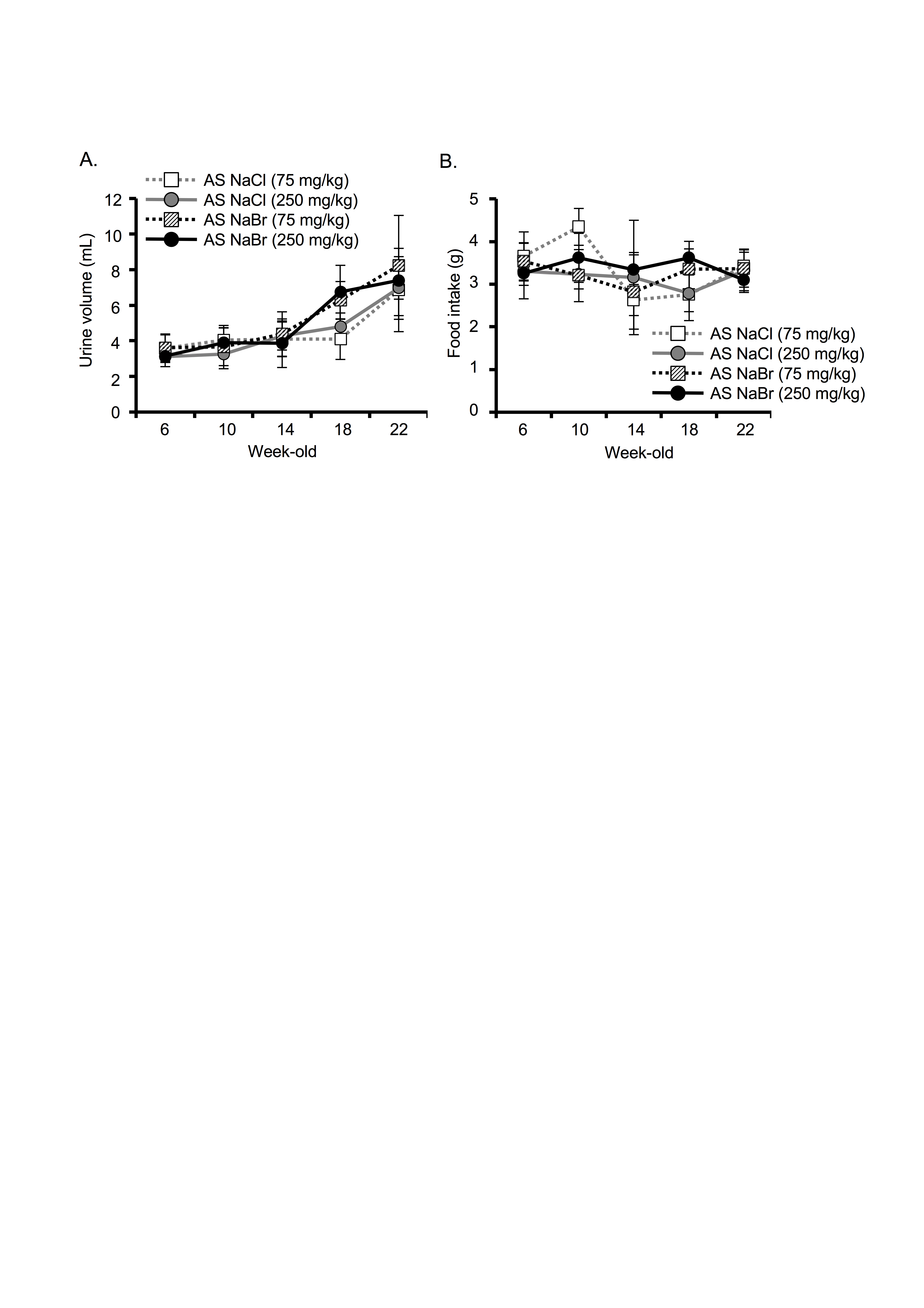

Supplement: S1 Fig — (A, B) Urine volume and food intake were measured every four weeks using metabolic cages for 24 hr. (TIF) [file pone.0183959.s001.tif]

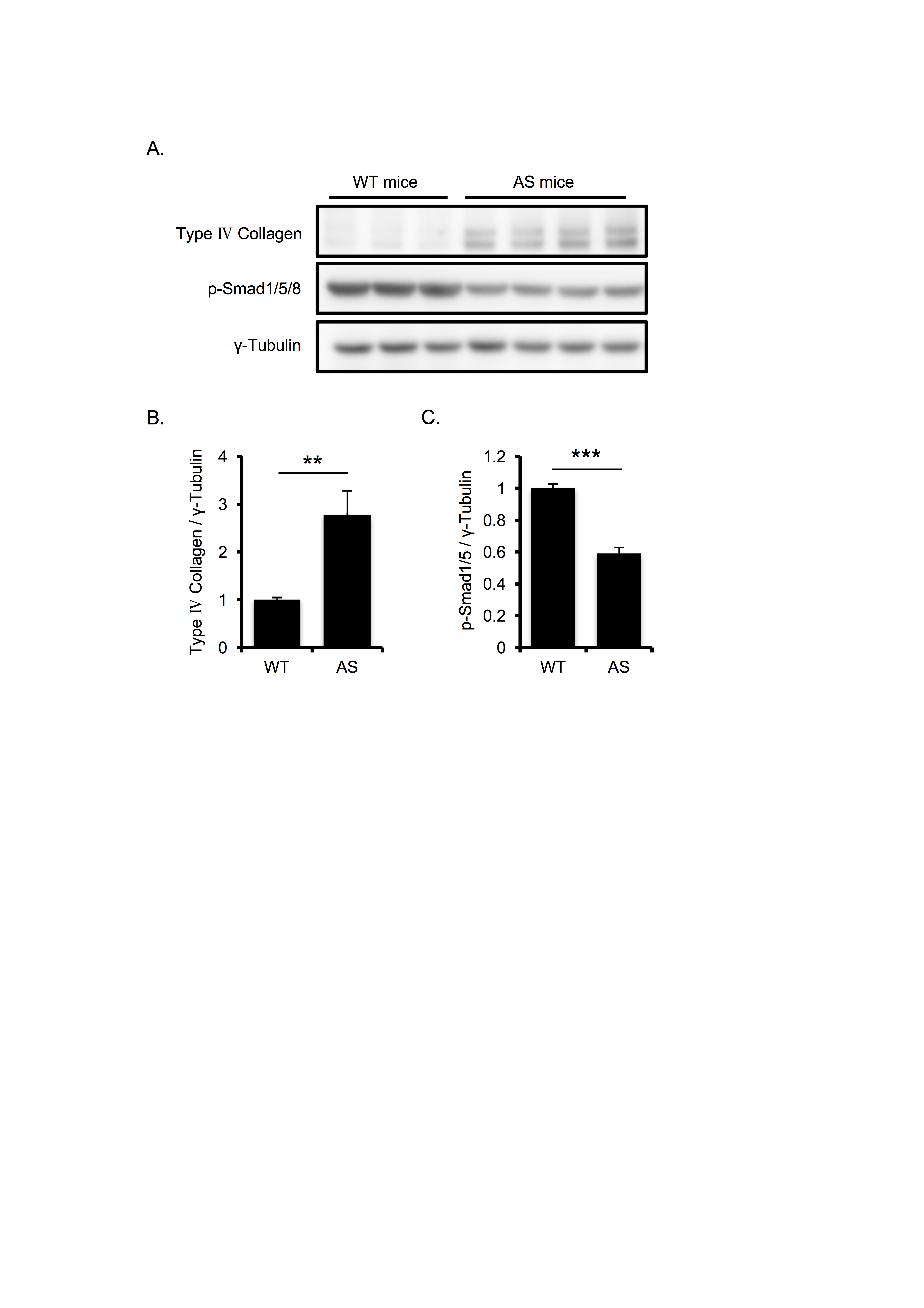

Supplement: S2 Fig — (A) Whole kidney protein lysates were isolated from 16-week-old WT and AS mice. Type IV collagen and phosphorylated Smad1/5/8 expression was analyzed by immunoblotting. (B) Immunoblots were quantified using Image Gauge software (Fujifilm), normalized to γ-tubulin and presented as relative expression. P values were assessed by unpaired t-test. (TIF) [file pone.0183959.s002.tif]

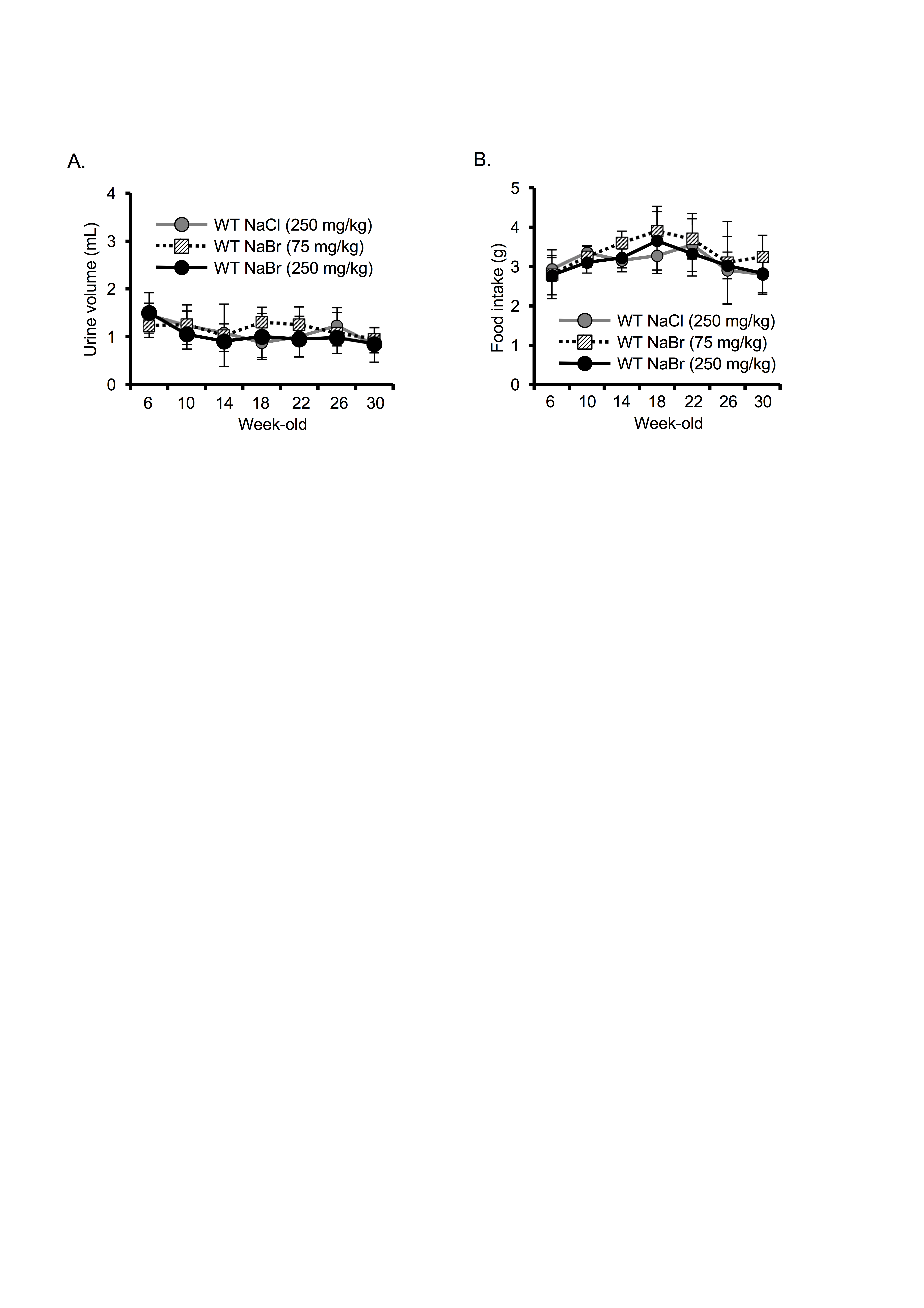

Supplement: S3 Fig — (A, B) Urine samples and food intake were measured every four weeks until mice were 30 weeks old. (TIF) [file pone.0183959.s003.tif]

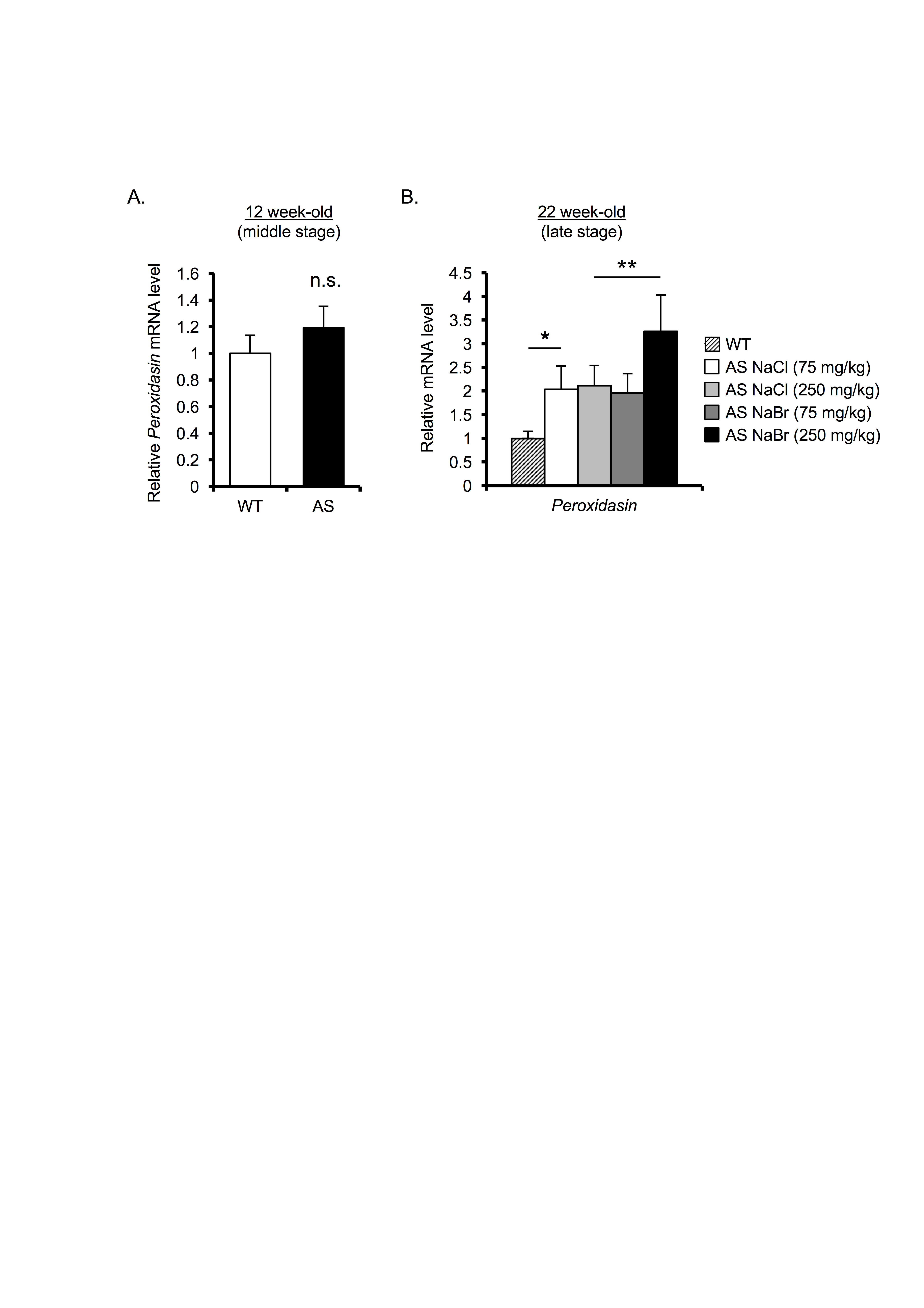

Supplement: S4 Fig — (A) Total RNA was isolated from renal tissues of 12-week-old (middle stage) WT and AS mice. Quantitative RT-PCR was performed to analyze the expression of Peroxidasin. (B) Total RNA was isolated from renal tissues of the indicated 22-week-old WT or (late stage) AS mice and quantitative RT-PCR analysis was performed. The data were normalized to Gapdh. Bars indicate the mean ± S.D. (n = 3–6). *P<0.05; **P<0.01, n.s., not significant. (TIF) [file pone.0183959.s004.tif]
